# Efficacy and Safety of Human Protein C Concentrate in Japanese Patients With Severe Congenital Protein C Deficiency

**DOI:** 10.1111/ped.70506

**Published:** 2026-09-15

**Authors:** Katsuyoshi Koh, Keiji Nogami, Harumi Kakuda, Yuri Okimoto, Moeko Hino, Chihiro Suzuki, Souhei Terashio, Hitoshi Ueda, Shouichi Ohga

**Affiliations:** ^1^ Department of Hematology/Oncology Saitama Children's Medical Center Saitama Japan; ^2^ Department of Pediatrics Nara Medical University Kashihara Japan; ^3^ Department of Hematology/Oncology Chiba Children's Hospital Chiba Japan; ^4^ Department of Pediatrics, Graduate School of Medicine Chiba University Chiba Japan; ^5^ Plasma‐Derived Therapies Strategy Unit, PDT R&D Takeda Pharmaceutical Company Limited Osaka Japan; ^6^ Japan Medical Office Takeda Pharmaceutical Company Limited Tokyo Japan; ^7^ Department of Pediatrics, Graduate School of Medical Sciences Kyushu University Fukuoka Japan

**Keywords:** clinical trial, protein C concentrate, protein C deficiency, purpura fulminans, SCPCD

## Abstract

**Background:**

Severe congenital protein C deficiency (SCPCD) is a rare disorder associated with life‐threatening thrombotic complications. Protein C concentrate is recommended for both acute and long‐term management of SCPCD. However, no efficacy and safety data in Japanese patients have been reported.

**Methods:**

In this extension phase of an open‐label, phase 1/2 clinical trial (ClinicalTrials.gov: NCT04984889), Japanese patients with SCPCD could receive intravenous protein C concentrate for on‐demand treatment, short‐term prophylaxis, or long‐term prophylaxis. Efficacy of on‐demand treatment was assessed using a predefined efficacy evaluation scale. For prophylaxis, efficacy was assessed based on the occurrence of purpura fulminans and thromboembolic complications during prophylaxis treatment. Safety was evaluated by recording adverse events (AEs).

**Results:**

All five patients who completed the pharmacokinetics phase of the study participated in this extension phase (mean [SD] age: 15.2 [8.9] years). At data cutoff, four patients had received eight on‐demand treatments of protein C concentrate for purpura fulminans; all were rated as effective. Protein C concentrate was administered for short‐term prophylaxis in one patient undergoing tooth extraction, with no thromboembolic complications observed. Six AEs were reported in three patients who received on‐demand treatment; none were treatment‐related. No AEs were observed during short‐term prophylaxis. There were no deaths or AEs leading to treatment discontinuation.

**Conclusions:**

Protein C concentrate was effective for on‐demand treatment and short‐term prophylaxis, and had an acceptable safety profile in this study population. These results support the use of protein C concentrate in the clinical management of patients with SCPCD in Japan.

**Trial Registration:**

ClinicalTrials.gov registration number: NCT04984889.

## 1 Introduction

Severe congenital protein C deficiency (SCPCD) is a rare autosomal recessive disorder with an estimated incidence of one in 250,000 to one in 4 million births [1, 2]. It is caused by homozygous or compound heterozygous pathogenic variants in the protein C gene (*PROC*), resulting in extremely low activity levels of protein C, a vitamin K‐dependent anticoagulant [1]. Severe protein C deficiency can cause life‐threatening thrombotic complications owing to the crucial role of protein C in regulating coagulation [1]. SCPCD usually manifests as purpura fulminans and disseminated intravascular coagulation within 72 h of birth and occasionally in later infancy [3, 4]. It is associated with high mortality and, given the discrepancy between the low number of patients diagnosed with SCPCD and the predicted incidence based on heterozygous protein C deficiencies occurring in one in 200–500 individuals, a high proportion of prenatal or neonatal deaths may occur before life‐saving therapy is administered [1, 5]. During the 5 years from 2014 to 2018, 190 patients were estimated to have neonatal thromboembolism (within 28 days of birth) in Japan, with a calculated incidence of 0.39 cases per 10,000 live births [6]. Nine patients in Japan were identified in that study to have homozygous, compound heterozygous, or heterozygous variants in *PROC* [6].

The 2022 recommendations on the diagnosis and management of SCPCD of the International Society on Thrombosis and Hemostasis include the use of plasma derived, viral‐inactivated protein C concentrate as the preferred option for the acute treatment of patients with SCPCD [4]. Protein C concentrate is also the recommended treatment option for both short‐term surgical prophylaxis and long‐term prophylaxis [4].

Plasma‐derived human protein C concentrate is indicated for prophylaxis and acute treatment of venous thrombosis and purpura fulminans in patients with SCPCD [7, 8]. However, despite the clinical use of protein C concentrate in the USA and Europe for acute treatment, short‐term prophylaxis, and long‐term prophylaxis in patients with SCPCD, data in Japanese patients are lacking [9]. The pharmacokinetics (PK) and safety of protein C concentrate for Japanese patients were evaluated in the first part (PK part) of a phase 1/2 open‐label, single‐dose, multicenter study, which included five asymptomatic Japanese patients with SCPCD. Protein C concentrate was well tolerated, and no serious treatment‐related adverse events (AEs) were reported. This extension part of the phase 1/2 study aimed to evaluate the efficacy and safety of protein C concentrate for on‐demand treatment, short‐term prophylaxis, and long‐term prophylaxis in Japanese patients with SCPCD.

## 2 Methods

### 2.1 Study Design

This is the extension phase of an open‐label, phase 1/2, non‐randomized, noncontrolled, multicenter study evaluating protein C concentrate (human) (TAK‐662) for the treatment of patients with SCPCD at four centers in Japan (ClinicalTrials.gov: NCT04984889). The overall study was initiated in September 2021, and the extension phase was completed on October 31, 2024. Here, we report the efficacy and safety findings from a prespecified interim analysis (data cutoff date: July 14, 2023). The study protocol and supporting study documents were approved by the Institutional Review Boards of Chiba University Hospital (approval number: 2021015), Nara Medical University Hospital (approval number: 21‐012), Saitama Prefectural Children’s Medical Center (approval number: 2021‐4) and Chiba Children’s Hospital (no approval number issued; approval date: 19 July 2021) before study initiation. This study was conducted in accordance with the Declaration of Helsinki, the International Council for Harmonisation of Technical Requirements for Pharmaceuticals for Human Use Good Clinical Practice Guidelines, and other applicable national and local ethical and legal requirements. All study participants, or their legal representative, provided informed consent before study initiation.

In the initial PK phase of the study, patients received a single intravenous dose of 80 IU/kg protein C concentrate. In the extension phase, protein C concentrate was to be provided for on‐demand treatment, short‐term prophylaxis, and/or long‐term prophylaxis at dosage ranges and treatment frequencies informed by previous clinical study data, overseas clinical practice, and the International Society on Thrombosis and Hemostasis guidelines for SCPCD [4, 5, 7]. Investigators were provided with participant PK data from the PK phase of the study, which could be used to inform dose adjustment at the investigator's discretion. All infusions of protein C concentrate were administered intravenously at a maximum injection rate of 2 mL/min, except for in children with a body weight of less than 10 kg, for whom the injection rate did not exceed a rate of 0.2 mL/kg/min. On‐demand treatment consisted of an initial infusion of 100–120 IU/kg protein C concentrate followed by three 60–80 IU/kg infusions every 6 h and subsequent 45–60 IU/kg infusions every 6 or 12 h until the resolution of non‐necrotic lesions and/or stabilization of thrombi. Short‐term prophylaxis for surgical procedures followed specific treatment regimens. For patients receiving anticoagulation therapy requiring elective (nonemergency) surgery, infusions of 100–120 IU/kg protein C concentrate once daily were initiated until anticoagulation therapy was successfully switched to protein C concentrate before surgery. For patients receiving anticoagulation therapy requiring emergency surgery, anticoagulation therapy was reversed with vitamin K supplementation, and 100–120 IU/kg protein C concentrate was initiated. In the 15 min before elective or emergency surgery, patients received an infusion of 60–80 IU/kg protein C concentrate, which was readministered every 6 h for the first 24 h. Between 24 and 48 h after surgery began, 45–60 IU/kg protein C concentrate was administered three times daily, after which 45–60 IU/kg protein C concentrate was administered twice daily. Long‐term prophylaxis was scheduled to consist of 45–60 IU/kg protein C concentrate administered twice daily, with dose adjustments at the investigator's discretion following measurement of protein C activity levels.

### 2.2 Patients

Patients were eligible for participation in the initial PK phase of the study if they had a diagnosis of SCPCD (having either homozygous or compound heterozygous *PROC* variants), were asymptomatic at study initiation, and were of Japanese nationality. Patients were eligible to continue to the extension phase if they had completed the PK phase of the study and if they developed acute purpura fulminans, warfarin‐induced skin necrosis, and/or other acute thromboembolic episodes diagnosed by the investigator as requiring on‐demand treatment. Patients were also eligible for the extension phase if they required protein C concentrate for short‐term prophylaxis for surgical procedures or for long‐term prophylaxis. Key exclusion criteria were the observation of any new serious medical conditions during the PK phase of the study that could have affected the patient's safety or treatment during the extension phase, and plans to participate in clinical trials for other investigational drugs or medical devices.

### 2.3 Efficacy Assessment

The severity of each purpura fulminans occurrence was determined based on the individual judgment of the investigator without pre‐established criteria. On‐demand treatment was rated by the investigator as “effective,” “effective with complications,” or “not effective” using an efficacy evaluation scale adapted from a US study of protein C concentrate in patients with SCPCD (Table 1) [5]. In situations in which short‐ or long‐term prophylaxis was provided, the occurrence of purpura fulminans or thromboembolic/thrombotic complications during prophylaxis treatment was recorded as an evaluation of efficacy.

**TABLE 1:** Efficacy evaluation scale for on‐demand treatment with protein C concentrate.

Rating	Definition
Effective	Stabilization or reduction of skin lesions, or stabilization of thrombi
Effective with complications	Effective with any of the following events: Adverse drug reactions leading to changes in dose or dosing frequency of protein C concentrate Adverse drug reactions leading to discontinuation of protein C concentrate Pathogenic virus infection
Not effective	Any other cases

### 2.4 Safety Assessment

AEs and serious AEs (SAEs) were evaluated from the time a patient first received protein C concentrate in the extension phase until the data cutoff date. AEs were coded using the Medical Dictionary for Regulatory Activities (MedDRA) version 25.0. Vital signs were measured before dosing for on‐demand treatment and before surgery, on day 2 of treatment and after surgery for short‐term prophylaxis.

### 2.5 Statistical Methods

Descriptive statistics were used to summarize efficacy and safety assessments for on‐demand therapy, short‐term prophylaxis, and long‐term prophylaxis.

## 3 Results

### 3.1 Patient Demographics and Characteristics

Five Japanese patients with SCPCD were enrolled in the study, all of whom completed the initial PK phase and were included in the extension. Patient demographic and baseline characteristics are shown in Table 2. The mean (standard deviation [SD]) age of the patients was 15.2 (8.9) years, and four of the patients were male. The median (range) duration of diagnosed protein C deficiency was 15.5 (3.3–27.4) years, and the median (range) protein C activity level at diagnosis was 10.0% (1.7–11.0). Four patients (three who received on‐demand treatment; one who received short‐term prophylaxis) received concomitant treatment with a vitamin K antagonist (warfarin) during the extension phase. One patient (who received short‐term prophylaxis) received 4‐factor prothrombin complex concentrate during the extension phase; however, this was administered after the period of short‐term prophylaxis with protein C concentrate was completed.

**TABLE 2:** Patient demographic and baseline clinical characteristics.

Characteristic	*n* = 5
Age, years	
Mean (SD)	15.2 (8.9)
Age category, *n* (%)	
< 10 years	1 (20)
10–19 years	2 (40)
20–30 years	2 (40)
Sex, *n* (%)	
Male	4 (80)
Female	1 (20)
Weight at screening^a^, kg	
Mean (SD)	34.0 (20.0)
Median (range)	20.5 (20.2–64.4)
Duration of diagnosed protein C deficiency, years	
Mean (SD)	15.39 (9.32)
Median (range)	15.5 (3.3–27.4)
Protein C activity level at diagnosis, %	
Mean (SD)	8.54 (3.85)
Median (range)	10.0 (1.7–11.0)
History of thrombotic complications/skin necrosis, *n* (%)	
Yes	5 (100)
Purpura fulminans	5 (100)
Coumarin‐induced skin necrosis/warfarin‐induced skin necrosis	0
Thrombotic complication	2 (40)
No	0
Previous treatment with blood products, *n* (%)	
Yes	2 (40)
No	3 (60)
Prophylactic treatment received, *n* (%)	
Yes	1 (20)
4‐factor prothrombin complex concentrate	1 (20)
No	4 (80)

Abbreviation: SD, standard deviation.

^a^ Refers to weight at screening before the start of the PK phase of the study.

### 3.2 Protein C Concentrate Administration

By the data cutoff for this analysis (July 14, 2023), four patients (age range: under 10 years to in their 20s) had received on‐demand treatment and one patient (aged in their 20s) had received short‐term prophylaxis with protein C concentrate (Table S1); no patients had received protein C concentrate for long‐term prophylaxis. Protein C concentrate was administered eight times for on‐demand treatment of purpura fulminans (once in one patient, twice in two patients, and three times in one patient) at a dose of 100–120 IU/kg. Protein C concentrate was administered 14 times over 7 days for short‐term prophylaxis in a patient undergoing surgical tooth extraction. This patient was previously receiving warfarin treatment as prophylaxis, which was discontinued 3 days before surgery to avoid excessive bleeding. The patient then received a single dose of 116.3 IU/kg protein C concentrate on the day before surgery, followed by four doses of 72.7 IU/kg on surgery days 1–2, then nine doses of 58.1 IU/kg from day 2 until the end of the 7‐day short‐term prophylaxis treatment period. Warfarin was restarted the day after surgery and continued to be administered while prophylaxis with protein C concentrate was ongoing.

### 3.3 Efficacy of Protein C Concentrate

All eight on‐demand treatments were for episodes of purpura fulminans and were rated “effective” using the efficacy evaluation scale (Table 3). Five of the purpura fulminans episodes were mild, and three were moderate. Four were first acute episodes, and four were second or later acute episodes. Overall, seven episodes required one dose of protein C concentrate, and one episode required two doses before symptom relief. The median number of doses per episode was one. The mean (SD) time to resolution was 2.0 (0.82) days for first acute episodes and 2.75 (2.22) days for second or later acute episodes. No purpura fulminans or thromboembolic complications were observed in the one patient who received short‐term prophylaxis.

**TABLE 3:** Efficacy evaluation for on‐demand treatment with protein C concentrate.

Assessment	On‐demand treatments^a^
Severity of purpura fulminans episode, number (%)	
Mild	5 (62.5)
Necrotic	1 (12.5)
Moderate	3 (37.5)
Necrotic	0 (0)
Treatment efficacy rating, number (%)	
Overall	
Effective	8 (100)
Effective with complications	0 (0)
Not effective	0 (0)
First acute episode	
Effective	4 (100)
Effective with complications	0 (0)
Not effective	0 (0)
Second and subsequent acute episodes	
Effective	4 (100)
Effective with complications	0 (0)
Not effective	0 (0)
Time to resolution in days, mean (SD)	
First acute episode	2.00 (0.816)
Second and subsequent acute episodes	2.75 (2.217)

Abbreviation: SD, standard deviation.

^a^ In total, eight on‐demand treatments were administered in four patients.

### 3.4 Safety Assessment

Overall, six AEs were reported in three patients who received on‐demand treatment (Table 4). All AEs were mild in severity, except for one SAE of respiratory failure. None of the AEs were considered treatment‐related. No AEs were observed in the one patient who received short‐term prophylaxis. No deaths or AEs leading to treatment or study discontinuation were reported. No clinically relevant changes in vital signs or clinically relevant abnormalities in laboratory assessments were observed.

**TABLE 4:** Summary of AEs in the extension phase.

AEs, *n* (%)	Patients (*n* = 5)
Any AE^a^	3 (60)
Infections and infestations	2 (40)
Nasopharyngitis	2 (40)
Oral herpes	1 (20)
Injury, poisoning, and procedural complications	1 (20)
Contusion	1 (20)
Respiratory, thoracic, and mediastinal disorders	1 (20)
Respiratory failure	1 (20)
Skin and subcutaneous tissue disorders	1 (20)
Decubitus ulcer	1 (20)
Any SAE	1 (20)
Respiratory, thoracic, and mediastinal disorders	1 (20)
Respiratory failure^b^	1 (20)

Abbreviations: AE, adverse event; SAE, serious adverse event.

^a^ All AEs were reported in patients who received on‐demand treatment; no AEs were reported in the patient who received short‐term prophylaxis.

^b^ This event occurred 196 days after the first protein C concentrate infusion in the extension phase, was resolved 16 days later, and was not considered treatment‐related.

## 4 Discussion

In the extension phase of this study, administration of protein C concentrate was effective for the on‐demand treatment of eight episodes of purpura fulminans in four Japanese patients with SCPCD. Protein C concentrate was also considered effective for short‐term prophylaxis in a patient requiring a surgical procedure, with no observations of purpura fulminans or thromboembolic complications. These results are consistent with the utility of protein C concentrate as a comprehensive formulation for both on‐demand treatment and prophylaxis in patients with SCPCD. Overall, six AEs were reported in three patients who received on‐demand treatment, none of which were considered to be related to treatment with protein C concentrate. Therefore, protein C concentrate appears to have an acceptable safety profile in this study population of Japanese patients with SCPCD.

Patients with SCPCD have a life‐long need for appropriate management options to prevent thrombotic complications [1, 4]. Protein C concentrate has so far not been available in Japan and, therefore, Japanese patients with SCPCD have been treated with fresh frozen plasma, activated protein C, and oral anticoagulants [10, 11, 12]. However, there are limitations to these approaches. In the neonatal period and in infancy, when SCPCD is typically most severe, there is a risk of both volume overload and inefficient protein C administration when fresh frozen plasma is used [13, 14]. Activated protein C carries an inherent risk of bleeding, and its short half‐life makes it unsuitable for prophylaxis [15]. There have also been cases in which it has been difficult to suppress thrombin formation using oral anticoagulants alone [15]. In the absence of approved long‐term prophylaxis options indicated for SCPCD in Japan, prothrombin complex concentrates containing protein C have also been used as an “off‐label” prophylaxis treatment alongside anticoagulants, with documented use in a small number of patients [15]. However, prothrombin complex concentrates also contain procoagulant factors, which may increase blood clotting risk. Overall, the efficacy of these treatments remains variable [15].

As of March 2024, protein C concentrate is now approved for use in Japan. The extension phase of this study enabled Japanese patients with SCPCD to receive protein C concentrate for on‐demand treatment, short‐term prophylaxis, or long‐term prophylaxis until the commercial protein C concentrate became available. If protein C concentrate is used for prophylactic administration, it is expected that administration to newborns suspected to have SCPCD (e.g., if they have an affected sibling) immediately after birth will reduce thrombotic events and help to avoid the development of sequelae. As of the data cutoff for the data presented here, no patients in the extension phase had received long‐term prophylaxis with protein C concentrate.

Overall, the results of this study provide clinically relevant data on the use of protein C concentrate for the management of SCPCD in Japan. Notably, the efficacy results of this study were consistent with that of an overseas report: there were no reported cases in which protein C concentrate was considered “not effective” for the treatment of acute episodes of purpura fulminans [5]. Given the rare occurrence of SCPCD, this study specifically used the same efficacy scale as the overseas report, in which the treatment could be rated as “effective,” “effective with complications” or “not effective.” Although the scale has limitations in being based on an older standard of SCPCD assessment and using more subjective rather than objective evaluation criteria, there are no other alternative scales available, and development of a new scale would present additional challenges regarding validation and assessment suitability. Additionally, as the goal of this study was to verify that the safety and efficacy of protein C concentrate in Japanese patients was consistent with overseas studies, it was considered most appropriate to use the same efficacy rating scale as the trial with the largest number of cases for ease of comparison.

Population PK modeling has also indicated that the PK of protein C concentrate is similar in Asian and non‐Asian patients [16]. PK parameters in Japanese patients upon protein C concentrate administration were investigated in the initial PK part of this study [17]. As in the initial PK phase of the study, protein C concentrate was well tolerated in Japanese patients, with no AEs leading to treatment or study discontinuation reported. Limitations of the current study include the small number of patients and that it was an extension of a non‐blinded, non‐randomized phase 1/2 study.

## 5 Conclusion

The results of the extension phase of this study demonstrate the efficacy and safety of protein C concentrate for on‐demand treatment and short‐term prophylaxis in Japanese patients with SCPCD. The study provides valuable treatment experiences regarding the administration of protein C concentrate in Japanese patients with SCPCD.

## Author Contributions

K.K., K.N., H.K., Y.O., M.H., S.T., H.U., and S.O. designed the study; K.K., K.N., H.K., Y.O., M.H., and S.O. executed the study, and K.K., K.N., H.K., Y.O., and S.O. acquired the data. All authors interpreted the data and read and approved the final manuscript.

## Funding

This work was supported by Takeda Pharmaceutical Company Limited.

## Conflicts of Interest

K.K. reports honoraria from Amgen, Bayer, Chugai Pharmaceutical, CSL, Fujimoto Pharmaceutical Corp, Japan Blood Products Organization, Nobelpharma, Novo Nordisk, Ohara Pharmaceutical Corp, Pfizer, Sanofi, Servier, and Takeda Pharmaceutical Company Limited. K.N. reports research grants from Bayer, Chugai Pharmaceutical, CSL, Fujimoto Pharmaceutical Corp, KM Biologics, Novo Nordisk, Sanofi, Sekisui, Sysmex, and Takeda Pharmaceutical Company Limited; and honoraria from Bayer, Chugai Pharmaceutical, CSL, Fujimoto Pharmaceutical Corp, KM Biologics, Novo Nordisk, Sanofi, Sekisui, Sysmex, and Takeda Pharmaceutical Company Limited. H.K. and S.O. report honoraria from Takeda Pharmaceutical Company Limited. Y.O. reports honoraria from Novo Nordisk and Takeda Pharmaceutical Company Limited. M.H. reports honoraria from Novo Nordisk, Sanofi and Takeda Pharmaceutical Company Limited; and patents planned, issued or pending with Novo Nordisk, Sanofi and Takeda Pharmaceutical Company Limited. C.S. and S.T. are employees and stockholders of Takeda Pharmaceutical Company Limited. H.U. was an employee and is a stockholder of Takeda Pharmaceutical Company Limited.

## Supporting information


**Table S1:** Summary of treatment episodes.

## Data Availability

Takeda does not plan to share data supporting the results reported in this article as there is a reasonable likelihood that individual patients could be reidentified due to the limited number of study participants.
