# Supplementary material for: Efficacy and Safety of Human Protein C Concentrate in Japanese Patients With Severe Congenital Protein C Deficiency
Source: Pediatr Int. 2026 Sep 15;68(1):e70506. doi: 10.1111/ped.70506 (PMC13579167; doi:10.1111/ped.70506)
Supplement: Supplementary file 1 [file PED-68-e70506-s001.pdf]

SUPPLEMENTARY TABLE

TABLE S1 Summary of treatment episodes.

Patient	Age	Protein C level at diagnosis (%)	Treatment episode	Concomitant oral anticoagulant	Treatment	Indication	Location	Necrotic (Yes/No)	Initial dose of PC concentrate (IU/kg)	Total dose of PC concentrate (IU/kg)	Time to resolution (days)
On-demand Treatment											
**Patient 1**	< 10	11	1	Warfarin	On demand	Purpura fulminans	Foot	No	100.0	100.0	3
			2	Warfarin	On demand	Purpura fulminans	Legs[a]	Yes	120.0	240.0	6
**Patient 3**	10–19	10	1	–	On demand	Purpura fulminans	Legs[a]	No	100.0	100.0	2
**Patient 4**	10–19	10	1	–	On demand	Purpura fulminans	Foot	No	100.0	100.0	2
			2	Warfarin	On demand	Purpura fulminans	Arm	No	103.0	103.0	2
			3	–	On demand	Purpura fulminans	Foot	No	110.0	110.0	1
**Patient 5**	20–30	1.7	1	Warfarin	On demand	Purpura fulminans	Leg	No	111.9	111.9	1
			2	Warfarin	On demand	Purpura fulminans	Thigh	No	111.9	111.9	2
Short term Prophylaxis											
**Patient 2**	20–30	10	1	Warfarin[b]	Short-term prophylaxis	Surgical tooth extraction	–	–	116.3	930.0	–

[a]These treatment episodes involved treatment of two lesions (left and right leg).
[b]This patient received warfarin concomitantly with protein C concentrate only after surgery.
